# *Plasmodium malariae* in Bangladesh

**DOI:** 10.1016/j.trstmh.2009.06.014

**Published:** 2010-01

**Authors:** W. Rahman, K. Chotivanich, K. Silamut, N. Tanomsing, A. Hossain, M.A. Faiz, A.M. Dondorp, R.J. Maude

**Affiliations:** a Chittagong Medical College Hospital, Chittagong, Bangladesh; b Mahidol-Oxford Tropical Medicine Research Unit, Faculty of Tropical Medicine, Mahidol University, 420/6 Rajvithi Road, Bangkok, 10400, Thailand; c Malaria Research Group (MRG), 1051/A, O.R Nizam Road, Mehdibag, Chittagong, Bangladesh; d Centre for Clinical Vaccinology and Tropical Medicine, Nuffield Department of Clinical Medicine, John Radcliffe Hospital, Oxford, OX3 7LJ, UK

**Keywords:** Malaria, severe, Plasmodium malariae, Rosetting, Bangladesh, PCR

## Abstract

We describe a 32-year-old Bangladeshi male presenting with severe malaria caused by a mono-infection with *Plasmodium malariae*. Rosetting of infected and uninfected erythrocytes, a putative virulence factor in falciparum malaria, was observed in the blood slide. Severe disease caused by *P. malariae* is extremely rare. The patient made a rapid recovery with intravenous quinine treatment.

A 32-year-old previously well Bangladeshi male labourer from Fatickchari in Chittagong District, Bangladesh, was admitted to Chittagong Medical College Hospital in July 2008 because of a 10-day history of evening fevers, chills, rigors, headache, anorexia, generalised myalgia, dry cough, palpitations, drowsiness and several generalised convulsions. He complained of abdominal pain and nausea without vomiting or diarrhoea for the preceding 5 days. He was treated initially with paracetamol at his local health complex, which controlled his fever. The day before admission he became disorientated and had generalized convulsions and was referred to Chittagong Medical College Hospital (CMCH) for further management.

On admission to CMCH, the patient was febrile (temperature 38.9 °C) and disorientated in time and place. His pulse rate was 110 beats per minute, blood pressure 95/55 mmHg, and respiratory rate 28 breaths per minute. Glasgow Coma Score was 14/15 (eyes 4/4, voice 4/5, movement 6/6). Physical examination was otherwise unremarkable. Haemoglobin, leukocyte count, blood glucose and blood biochemistry, including serum creatinine, were normal and he had a mild thrombocytopaenia of 93 000 per mm^3^.

A thin blood film showed *P. malariae* with a parasitaemia of 3 per 1000 red cells (14 017/uL), with typical band form trophozoites, schizonts containing 4-6 merozoites, and occasional gametocytes. Rosettes were observed in a frequency of 50 per 100 trophozoite-infected red cells (Figure 1). Rapid PfHRP2-based antigen testing was negative for *P. falciparum* (Paracheck, Orchid Biomedical Systems, Goa, India). Polymerase chain reaction (nested PCR based on the 18 s rRNA gene) performed on the same blood sample subsequently confirmed a diagnosis of *Plasmodium malariae* malaria, whereas the PCRs for *P. falciparum*, *P. vivax*, *P. ovale* and *P. knowlesi* were negative (Supplementary file 1).

**Figure 1:**
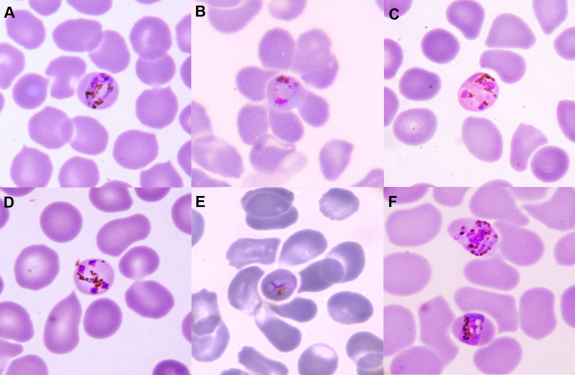
Light microscopy of *P. malariae* infection in thin peripheral blood film, 1000x magnification, photographed through the microscope eyepiece using a Canon IXUS 860IS compact digital camera.^7^**A.** trophozoite **B.** trophozoite with surrounding rosette **C.** schizont **D.** schizont **E.** trophozoite with surrounding rosette **F.** two trophozoites including one band form.

As the patient was prostrated and had multiple convulsions he was treated as having severe malaria with a loading dose of intravenous quinine (parenteral artesunate is not yet registered in Bangladesh). The patient received a total of 4 doses of intravenous quinine followed by further oral treatment and made a rapid recovery. He was afebrile after 3 days and discharged himself from hospital.

*P. malariae* malaria has been reported rarely from Bangladesh.^1^ Early stages of *P. malariae* can be difficult to distinguish from other species by microscopy alone and its prevalence may be underestimated. This is the first PCR-confirmed case of *P. malariae* infection to be reported from Bangladesh. The patient was remarkable because of his presentation with neurological features (prostration and multiple convulsions) compatible with a diagnosis of severe malaria in accordance with World Health Organization criteria.^2^ Severe malaria is an extremely rare manifestation of a monoinfection with *P. malariae*.^3^ The classic quaternary pattern of fever every 72 h was absent, indicating a lack of synchronicity of the infection.

This patient showed rosette formation in the peripheral blood slide, defined as the binding of two or more infected red cells to an uninfected red cell.^4^ Although rosetting has been reported in infections with *P. vivax*, *P. ovale* and *P. malariae*,^5^ it is usually described only in *P. falciparum*^4^ after in-vitro maturation of the parasites. It has been associated with microcirculatory blood flow obstruction and severe disease, although this is controversial.^6^ Rosetting of *P. falciparum* is thought to be mediated by binding of various erythrocyte surface cell receptors to the surface expressed variant protein PfEMP1. The mechanism in the other three species is unknown. It is highly uncertain whether in *P. malariae* rosettes significantly obstruct microvascular flow, since parasitaemia is invariably low. Further studies will be needed to establish their resistance to shear forces and the relationship with disease severity in *P. malariae*. Usual treatment of uncomplicated *P. malariae* infection is with chloroquine or an artemisinin-based combination therapy.^2^ However, because of the disease severity, this patient was treated with intravenous quinine.

In summary, we report a rare case of a PCR-confirmed monoinfection with *P. malariae* presenting with severe disease and rosetting in the peripheral blood film. The patient made a quick recovery with intravenous quinine.  

## Authors' Contributions

All authors contributed to the conception and content of this case report. WR carried out the clinical assessment. RJM took the photographs. NT did the PCR. RJM, WR, KC, KS, NT, AH, MAF and AMD drafted and revised the manuscript. RJM, NT, KC and KS prepared the figures. All authors read and approved the final manuscript.  

## Funding

Mahidol-Oxford Tropical Medicine Research Unit is funded by the Wellcome Trust of Great Britain.  

## Conflicts of Interest

None declared.  

## Ethical approval

Ethical clearance was obtained from the Oxford Tropical Research Ethics Committee (OXTREC) and the Bangladesh Medical Research Council. The patient gave informed written consent.

## Guarantor

AMD is guarantor of the paper

## Appendix A Supplementary data

Supplementary File 1 Results of agarose gel analysis of nested PCR for species-specific detection of Plasmodium DNA, based on the 18 s rRNA gene. S = molecular base pair standard, F = falciparum, V = vivax, M = malariae, O = ovale and K = knowlesi. The diagnostic band for *P. malariae* is circled in white. Bands for Pm and Pv in a control sample with mixed infection are indicated by white arrows.
