# Supplementary figures and images for: Plasmodium malariae in Bangladesh
Source: Trans R Soc Trop Med Hyg. 2010 Jan;104(1):78–80. doi: 10.1016/j.trstmh.2009.06.014 (PMC2793369; doi:10.1016/j.trstmh.2009.06.014)

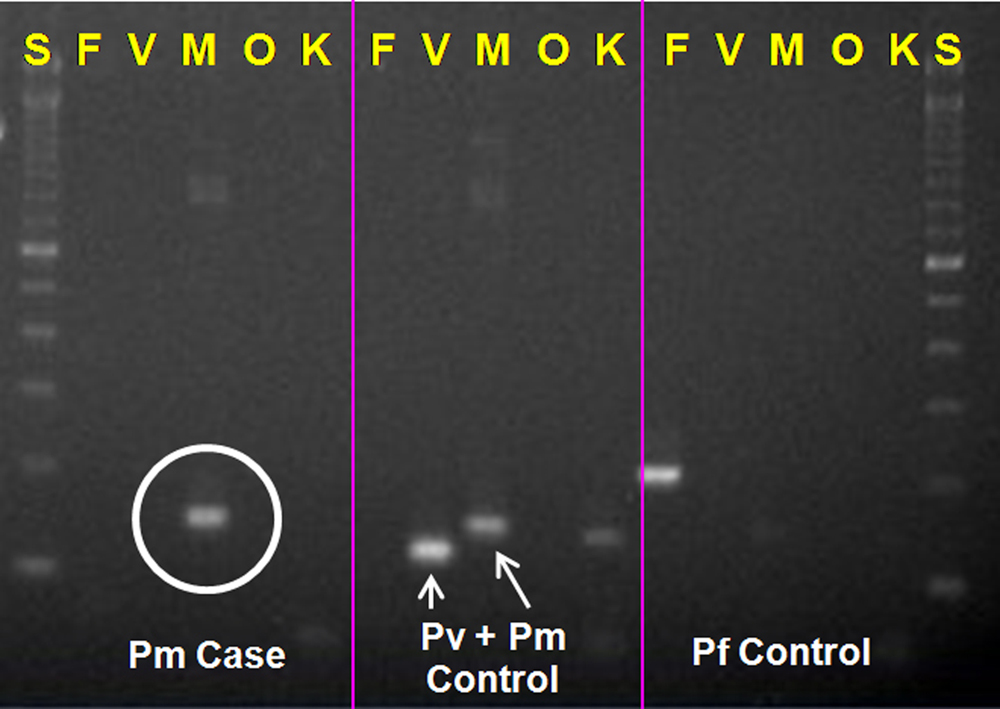

Supplement: Supplementary File 1 — Results of agarose gel analysis of nested PCR for species-specific detection of Plasmodium DNA, based on the 18 s rRNA gene. S = molecular base pair standard, F = falciparum, V = vivax, M = malariae, O = ovale and K = knowlesi. The diagnostic band for P. malariae is circled in white. Bands for Pm and Pv in a control sample with mixed infection are indicated by white arrows. [file mmc1.jpg]
